# UNDERSTANDING THE COMPLEXITY AND USE CHALLENGES OF MEDICARE PLAN FINDER FOR OLDER ADULTS

**DOI:** 10.1093/geroni/igad104.2824

**Published:** 2023-12-21

**Authors:** Hye Soo Lee, Maurita Harris, Wendy Rogers

**Affiliations:** University of Illinois--Urbana-Champaign, Champaign, Illinois, United States; Wilfrid Laurier University, Brantford, Ontario, Canada; University of Illinois Urbana-Champaign, Champaign, Illinois, United States

## Abstract

It is critical for older adults to find an affordable health plan that meets their needs. The Medicare.gov site provides a Medicare plan finder (MPF), which is an online tool designed to assist individuals to find a Medicare plan that is most suitable for them. The MPF was updated in 2021 but remains complex and may be especially difficult for older adults to use, given age-related changes in cognitive abilities. To identify potential use challenges for MFP, we documented the complexity of the steps required for accomplishing the goal of finding a plan. We completed a task analysis using two use-case scenarios to illustrate potential paths one could take in using MPF. Both use cases were set to enroll in the same plan to explore how and why their paths could differ, focusing on whether they have prior information and experience with Medicare and MPF. The number of steps taken by these two use cases ranged from 10 steps (experienced user) to 32 steps (novice user), due to the different needs they had for using MPF. The results imply that the one-for-all design of MPF may not be optimal for meeting the different needs of users. We propose two solutions to reduce the number of steps for using MPF. First, users need to be able to access the functions they want from the start (e.g., using quick links from the initial page). Second, users would benefit from learning what they need to know and prepare before they start using MPF.

